# Pain Complaints and Intubation Risk in COVID-19 Patients: A Retrospective Cross-Sectional Analysis

**DOI:** 10.7759/cureus.81585

**Published:** 2025-04-01

**Authors:** Michael Reimer, Victor Lee, Oluseyi Obadeyi, Kaitlyn S Reimer, Christopher Tarver, Duc A Tran, Eugene Pak

**Affiliations:** 1 Physical Medicine and Rehabilitation, University of Michigan, Ann Arbor, USA; 2 Emergency Medicine, Loma Linda University Medical Center, Loma Linda, USA; 3 Pain Medicine, Loma Linda University Medical Center, Loma Linda, USA; 4 Psychiatry, University of Michigan, Ann Arbor, USA; 5 Physical Medicine and Rehabilitation, Loma Linda University Medical Center, Loma Linda, USA

**Keywords:** abdominal pain, chest pain, covid, covid-19, headache, hospital, intubation, myalgia, pain, sars-cov-2

## Abstract

Purpose

In late 2019, the SARS-CoV-2 virus spread to become a worldwide pandemic with continued impact today. The disease severity is categorized based on age, comorbid conditions, and respiratory symptoms, but the clinical significance of pain reports and their correlation with life-sustaining treatment is not addressed much in the literature. The purpose of this study is to investigate the relationship between pain reports in patients with COVID-19 and the likelihood of intubation.

Methods

A retrospective cross-sectional analysis was performed compiling representative billing codes for pain complaints using the Epic Cosmos data set, a HIPAA limited data set of more than 226 million patients from 236 health systems using Epic software. For validation of this method, three months of institutional-specific Cosmos billing code data were compared to chart-reviewed pain complaints at a university health system. After validation, the data was broadened to include the entire Cosmos database from February 1, 2020 through April 15, 2023 for patients with confirmed COVID-19 infections seen in emergency rooms or an inpatient ward. Using billing codes, these patients were divided into different pain groups: abdominal pain, chest pain, myalgia, headache, or none of these, and then further subdivided based on intubation status. Lastly, patients were divided by age: <18 years, 18 to <40 years, 40 to <50 years, 50 to 65 years, and ≥65 years of age. Pearson's chi-square analyses were performed to investigate the relationship between pain symptoms and intubation and further performed to assess variations by age subgroup. Odds ratios (ORs) of the data were calculated in the same manner.

Results

We investigated 2,491,770 data points in our analysis of COVID-19 positive emergency room and inpatient cases. When comparing the presence of all pain types combined and intubation by the chi-square test, the p-value (p) was <0.00001, suggesting that a relationship exists between pain and intubation. OR analysis showed that those with pain complaints were less likely to be intubated with OR 0.58 (95% confidence interval (CI) 0.57 to 0.59, p <0.0001). Pain subgroups similarly showed reduced likelihood of intubation: abdominal pain, OR of 0.61 (95% Cl: 0.59 - 0.62), chest pain, OR of 0.86 (95% Cl: 0.85 - 0.88), myalgia, OR of 0.26 (95% Cl: 0.24 - 0.27) and headache, OR of 0.31 (95% CI: 0.29 - 0.32).

Conclusions

Due to the wide variability in COVID-19 infection symptoms, it is difficult to identify a single risk factor that correlates with an increased likelihood of intubation. Our cross-sectional examination of 2.4 million data points for COVID-19 patients in the emergency department or inpatient settings found that pain complaints were negatively correlated with emergency intubation. Given this, the absence of pain reports in hospitalized COVID-19 patients may imply poor prognosis; however, further research is needed to determine whether the absence of pain is indeed a poor prognostic indicator and if so, hospitalized COVID-19 patients without pain warrant close monitoring.

## Introduction

With the novel 2019 SARS-CoV-2 virus emergence, subsequent pandemic, and recurrent viral mutations, the world has been impacted with over 700 million cases reported worldwide [1,2]. Research focused on categorizing disease severity by age, co-morbid conditions, and respiratory symptoms [3-7]. There are cross-sectional studies examining the worsening of acute, chronic, and post-surgical pain due to the COVID-19 pandemic, in part associated with care access limitations and psychological factors such as fear of infection, worsening mental conditions including anxiety and depression, and generalized pandemic-related stress [8-11]. Alternatively, some case reports suggest that COVID-19 infection may interfere with nociception, resulting in chronic pain resolution during active infection [12]. However, studies directly comparing COVID-related pain and disease severity, including correlation with life-sustaining treatment such as intubation, are limited [13]. 

Because of COVID-19’s increase in patient hospitalizations, complications to other medical care, and effect on vulnerable populations, it is important to further study the virus and its effects to prepare for future resurgences [2,6,9,14]. The objective of this study is to examine the relationship between patients with COVID-19 reporting pain and the likelihood of intubation during their hospital stay. The null hypothesis was that patients with COVID-19 in a hospital setting who lacked pain complaints were equally as likely to be intubated as patients who had pain complaints.

## Materials and methods

A retrospective cross-sectional analysis was performed using ICD-10 symptom and diagnostic billing codes (Tables 6-9) from the Epic Cosmos data set. Cosmos is a HIPAA limited data set of more than 226 million patients from 236 health systems that use Epic software across the United States, representing all 50 states [15]. 

Within the Cosmos system, patient encounters were selected using the following inclusion criteria: patients seen in the emergency room or inpatient setting, where they also tested positive for COVID-19 by hospital laboratory. This data was divided into five groups: those with abdominal pain, chest pain, myalgia, headache, and none of the above. Validation of billing code data as a metric for pain complaints was performed via side-by-side comparison of Cosmos data for an academic medical center from February 1, 2020 through April 27, 2020 with prior hand chart-reviewed pain data from the same time period and institution (Table 1) [13]. 

**Table 1:** COVID-19 Pain Complaints per Chart-Review Versus Cosmos Billing Codes at Home Institution From February 1, 2020 through April 27, 2020

	Chest Pain	Abdominal Pain	Headache Pain	Myalgia
Cosmos	25	17	12	<10
Chart-review	24	16	14	22

After comparison of Cosmos billing code data to chart-reviewed pain complaints, the Cosmos data set was expanded to include all available patient encounters from participating health systems across the United States from February 1, 2020 through April 15, 2023. Patient data within Cosmos was grouped into those who were intubated and those who were not and further divided by age: < 18 years, 18 to < 40 years, 40 to < 50 years, 50 to 65 years, and ≥ 65 years of age prior to extraction into Microsoft Excel (Microsoft Corporation, Redmond, USA). The entirety of the dataset used for this project was de-identified: either by Cosmos or by the prior research team [13]. For this reason, this research was deemed Institutional Review Board exempt.

Using Microsoft Excel, these data underwent Pearson's chi-square analyses comparing the presence of all pain symptoms with intubation, sub-groups for pain types with intubation, and finally comparison of sub-groups for patient age with intubation. Odds ratio (OR) analyses were performed in the same manner.

## Results

In our sample of COVID-19 positive emergency room or inpatient encounters, there were a total of 1,307,321 with pain complaints and 1,184,449 without pain complaints for a total of 2,491,770 data points. Of these data points, 62,321 were intubated and 2,429,449 were not intubated. According to Cosmos [15], patient demographics are a representative sample of patients in the United States with close alignment to U.S. Census Bureau Data. The age distribution of the sample and pain type distribution can be seen in Table 2 and Table 3.

**Table 2:** Age Range Distribution of Sample Age ranges were split into five groups as outlined in the top column. Percentages shown represent the amount of data from each age group.

Age Group					
	< 18	18 to < 40	40 to < 50	50 to < 65	≥ 65	All Ages
# of Data Points	248,492	607,574	282,122	513,468	840,114	2,491,770
% of Total Data	10.00%	24.40%	11.30%	20.60%	33.70%	100%

**Table 3:** Pain Type Distribution of Sample Pain type was divided into five groups as outlined in the top column. Percentages shown represent the amount of data from each pain group.

Abdominal Pain	Chest Pain	Myalgia	Headache	No Pain	Total
351,707	475,950	161,893	317,771	1,184,449	2,491,770
14.1%	19.1%	6.5%	12.8%	47.5%	100%

Combined pain types

**Figure 1:**
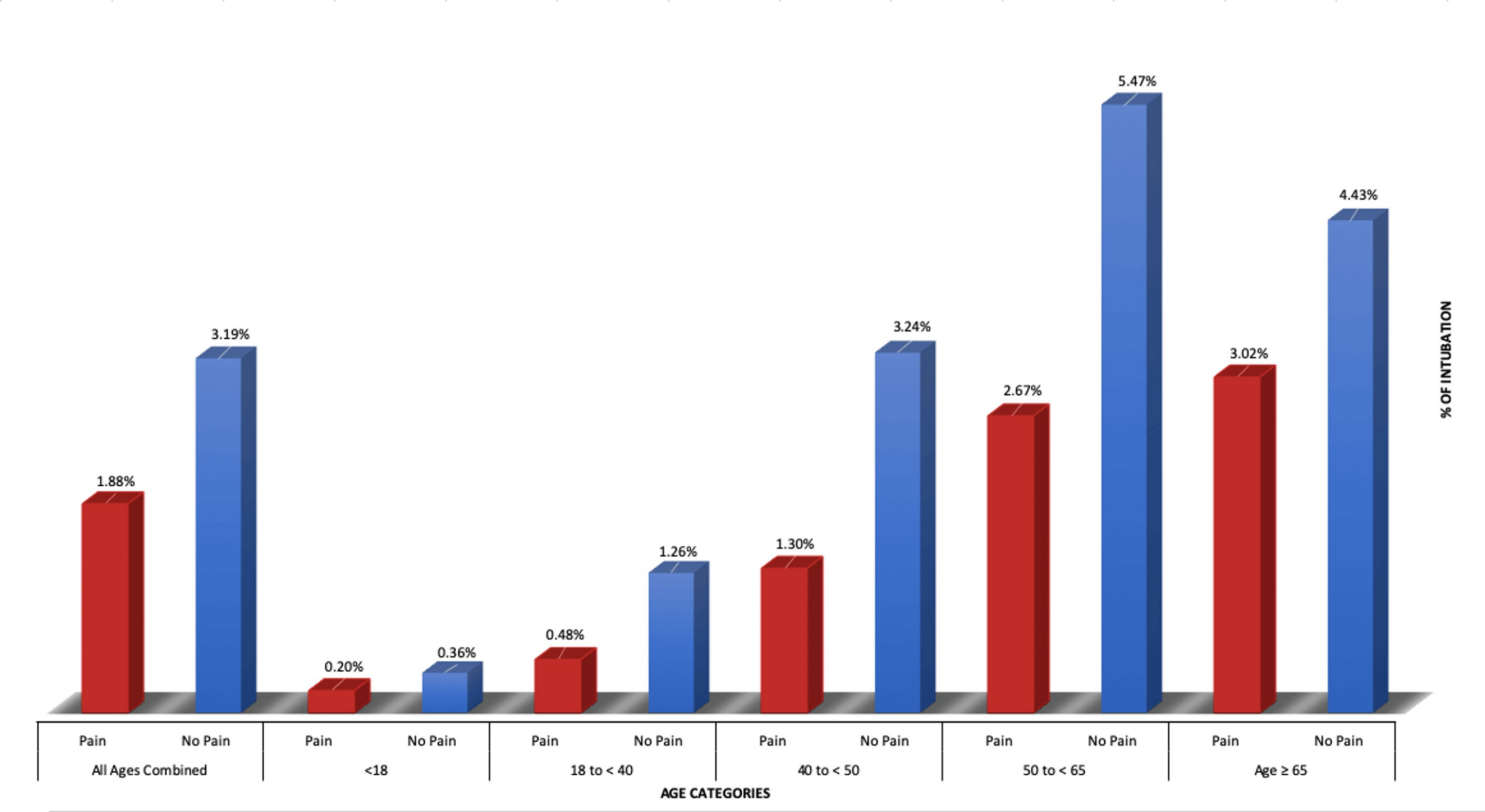
Intubation by Age for Combined Pain Types

With the above sample, there was a significant association by the chi-square test between pain (all types) and intubation (p <0.00001). This was consistent for all age subgroups (Figure 1).

An OR of 0.58 (95% confidence interval (CI): 0.57-0.59, p <0.0001) indicates that pain complaints were significantly associated with lower odds of intubation in hospital encounters for patients with COVID-19 (Table 4). 

**Table 4:** Odds Ratio of Having Pain Complaints and Being Intubated Delineated by Age Group

Ages	Odds Ratio	95% CI
All ages combined	0.58*	0.57 - 0.59
<18	0.54*	0.45 - 0.67
18-<40	0.38*	0.36 - 0.40
40-<50	0.39*	0.37 - 0.42
50-<65	0.47*	0.46 - 0.49
>65	0.67*	0.66 - 0.69
*Significant at p < 0.0001		

**Figure 2:**
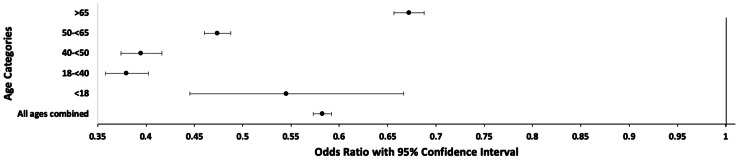
Odds Ratio of Having Pain Complaints and Being Intubated by Age Group

When all pain types were broken down into age subgroups, each subgroup by OR analysis showed that pain was negatively associated with intubation at a 95% CI (Table 4, Figure 2).

Abdominal pain

**Figure 3:**
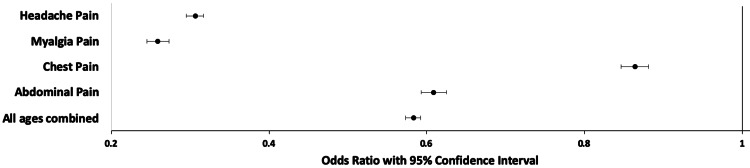
Odds Ratio of Pain Type and Being Intubated

When comparing the presence of abdominal pain complaints and intubation by the chi-square test, the p-value was <0.00001. This was consistent for all age groups with the exception of the <18 years old group which was not significant at p <0.01. An OR of 0.61 (95% CI: 0.59 - 0.62, p <0.0001) indicates that abdominal pain complaints were associated with lower odds of intubation (Table 5, Figure 3). 

**Table 5:** Odds Ratio of Pain Type and Being Intubated

Type of Pain	Odds Ratio	95% CI
Abdominal Pain	0.61*	0.59 - 0.62
Chest Pain	0.86*	0.85 - 0.88
Myalgia Pain	0.26*	0.24 - 0.27
Headache Pain	0.31*	0.29 - 0.32
All Pain Types	0.58*	0.57 - 0.59
*Significant at p < 0.0001		

Chest pain

When comparing the presence of chest pain complaints and intubation by the chi-square test, the p-value was <0.00001. This was consistent for all age groups with the exception of the <18 years old group, which was not significant at p <0.01. An OR of 0.86 (95% Cl: 0.85 - 0.88, p <0.0001) indicates chest pain complaints were associated with lower odds of intubation (Table 5, Figure 3). 

Myalgia

When comparing the presence of myalgia pain and intubation by the chi-square test, the p-value was <0.00001. This finding was consistent for all age groups with the exception of the <18 years group, where a similar trend was identified, which did not reach statistical significance. An OR of 0.26 (95% Cl: 0.24 - 0.27, p <0.0001) indicates that myalgia pain complaints were associated with lower odds of intubation (Table 5, Figure 3). 

Head pain or headache

Lastly, when comparing the presence of head pain or headache complaints and intubation by the chi-square test, the p-value was <0.00001. This was consistent for all age groups. An OR of 0.31 (95% Cl: 0.29 - 0.32, p <0.0001) indicates that headache or head pain complaints were associated with lower odds of intubation (Table 5, Figure 3).

## Discussion

This study adds to the body of knowledge addressing the association between COVID-19 patient experiences and their outcomes. Many COVID-19 investigations have focused on the common symptoms of COVID-19, such as dyspnea, fever, and cough [2,4,5,16]. However, there is less known about COVID-19's atypical presenting symptoms including pain manifestations [10,13]. 

Our study sought to determine if pain in COVID-19 infections was associated with increased risk of intubation. According to our findings, with the exception of those with abdominal and chest pain under <18 years old, pain is negatively associated with intubation in patients with COVID-19. A prior study at our institution with a small sample size found that patients with COVID-19 infection and abdominal pain (n=16, 6 intubated, 10 non-intubated) were positively associated with intubation when compared to patients with COVID-19 who did not have abdominal pain [13]. We believed a larger sample might re-demonstrate those findings. Our study did reject the null hypothesis that there was no difference in the intubation rates for patients with COVID-19 in a hospital setting who did and did not have pain. However, the results of this study conversely suggest that pain complaints are negatively associated with intubation, OR 0.58 (Table 5). In our results, differing pain groups had comparatively lower risk of being intubated, with the myalgia subgroup (OR 0.26) having the lowest risk of intubation, followed by head pain (OR 0.31). 

One plausible explanation of our results is that patients with severe COVID-19 infections who subsequently required intubation may have been unable to report pain complaints due to delirium or altered mental status associated with the severity of their illness. Patients requiring intubation are often either unable to protect their airway or experiencing hypoxic or hypercapnic respiratory failure, which could affect a patient’s ability to report pain complaints. Alternatively, COVID-19 has diverse effects on pain expression, and it is possible that severe COVID-19 infections may be associated with decreased nociception [12]. One study found that COVID-19 spike protein interaction with vascular endothelial growth factor-A (VEGF-A) and neuropilin-1 receptors produces analgesia [17].

Our sample again demonstrated that a variety of symptomatology accompanies COVID-19 infections [2,4-7,10,18,19]. Fifty-two percent of data points in our study (from total n=2.49 million) exhibited at least one of the four types of pain assessed, re-demonstrating that pain is a common complaint associated with COVID-19. Chest pain (19.1%) and abdominal pain (14.1%) were the most common pain types in our study. The precise mechanism by which COVID-19 may cause diverse pain complaints is multifaceted; its interaction with angiotensin-converting enzyme 2 receptors and increasing circulating cytokine levels have been implicated in COVID-19 infection-induced pain [11], whereas aforementioned spike protein interactions can also have an analgesic effect [17]. These differing mechanisms may provide insight into why some COVID-19 patients may have no pain, as observed in our study, or even instances of chronic pain relief [12]. 

Similar to prior studies [3,6,11,20-23], age appeared to play a substantial role in illness severity. One meta-analysis of more than 600,000 COVID-19-infected patients from diverse countries revealed an age-related mortality increase [21]. During COVID-19-related admissions, older age is related to an increase in the Sequential Organ Failure Assessment (SOFA) score and a D-dimer larger than 1 g/mL, which has been linked to increased mortality [24]. This increase in COVID-19 severity is likely due to the increase in comorbid medical conditions with age [7,11,12,23,25]. This was again demonstrated in our study, with the “50 to <65” and “≥65” age groups having the highest intubation rates (Figure 1). Increased age was generally associated with increased ORs for intubation. The OR for age group 18-40 was 0.38, that for age group 40-50 was 0.39, that for age group 50-60 was 0.47, and that for age group > 65 was 0.67. The OR for the age group <18 did not follow this trend and was 0.54. This may be attributable to the smaller sample size in the <18 age group, or alternatively differing severity of disease processes for the pediatric population when seen in a hospital setting with COVID-19 infection. This study highlights the significant correlation between specific pain symptoms and a reduced risk of intubation while also pointing out age-related trends with intubation, which is associated with severe COVID-19 infections.

The study was not without multiple limitations. The study design is cross-sectional for a period of a patient's hospital encounter, meaning that during a patient's hospitalization, pain complaints were assessed and compared with the occurrence of intubation. A retrospective cohort of patients with COVID-19 would better evaluate the timing of pain complaints, or lack thereof, and progression to intubation. The Cosmos database has easily accessible representative data for the United States of America; however, one of the major limitations of Cosmos is that it provides aggregate, not individual, data. With the data set, it was not possible to do a retrospective cohort study, assess the severity of individual's COVID-19 infections using a graded scale, or control for comorbid medical conditions in the sample. Furthermore, we could not assess patients' primary reason for hospital encounter, only that they had COVID-19 and were seen in the hospital between February 1, 2020 and April 15, 2023 which occurred during the COVID-19 pandemic. 

Additionally, the Epic Cosmos patient database provided over 2.4 million data points for the study; the size of this data was cumbersome to our computing capacity; thus, we had to pull data in smaller quantities in quarterly chunks. Because younger patients visiting the emergency department or admitted with COVID-19 were a smaller subset in the study, when the data was parsed into different pain categories, particularly head pain and myalgia, for some quarters there were fewer than 10 data points in those groups, which Cosmos displayed as "<10". From the total pain complaints for a particular quarter, this number could typically be ascertained by subtracting each subgroup from the total data points for the quarter. However, on the rare occasion that two age groups had less than 10 data points, the numbers represented by the <10 value in Cosmos were valued at zero for our calculations. Given the negative correlation of the data, this presents a possible confounding factor. However, given the sample size of the 2.4 million data points and the rarity of this occurrence, this is unlikely to affect the statistical outcomes. 

This research sample should be generally reproducible; however, the Cosmos database is constantly growing, so exact reproduction of our data is unlikely. In Cosmos’ data sorting tool, pain was pulled as a single complaint, i.e., chest pain, and examined independently. Thus, the research strictly examined complaints, rather than patients, and their associated outcomes. When running chi-square calculations, abdominal pain was compared to the group without any pain complaints. A comparison alternatively could have been done between those with one pain complaint and all other data points (other pain types and no pain complaints). This however could have compared multiple data points from the same patient should they have had multiple pain-type complaints; therefore, this was not chosen for our study design. 

Lastly, our data validation showed great concordance between chart and hand-reviewed data for headache, chest pain, and abdominal pain; however, the data for myalgia left room to be desired (Table 1). The billing code used for that complaint pulled a smaller group than the hand-reviewed data group. When extrapolated to the full Cosmos sample, this group again remained small (Table 3) and was also the most negatively correlated with intubation. Although this sample is likely smaller than the true myalgia population, it may represent a more accurate sampling of myalgia as the three billing codes (Table 8) were specific to myalgia. 

## Conclusions

Due to the wide variability in COVID-19 infection symptoms, it is difficult to identify a single risk factor that correlates with an increased likelihood of intubation. Our cross-sectional examination of data points for COVID-19 patients in the emergency department or inpatient settings found that pain complaints were negatively correlated with emergency intubation. Given this, the absence of pain reports in hospitalized COVID-19 patients may imply poor prognosis; however, further research is needed to determine whether the absence of pain is truly a poor prognostic indicator; if so, hospitalized COVID-19 patients without pain warrant close monitoring.

## Appendices

Below are the ICD-10-CM Billing Codes Used to Compile Cosmos Pain Sub-Groups. 

**Table 6:** ICD-10-CM Billing Codes Used to Compile Cosmos Abdominal Pain Sub-Group

ICD-10-CM Code	Code Description
R10.8	Other abdominal pain
R10.9	Unspecified abdominal pain
R10.84	Generalized abdominal pain
R10.30	Lower abdominal pain, unspecified
R10.10	Upper abdominal pain, unspecified
R10	Abdominal and pelvic pain
R10.*	Abdominal and pelvic pain
R10.81	Abdominal tenderness

**Table 7:** ICD-10-CM Billing Codes Used to Compile Cosmos Chest Pain Sub-Group Chest pain includes 676 ICD-10-CM codes grouped together by Cosmos. Several representative codes are listed above.

ICD-10-CM Code	Code Description
R07.9	Chest pain, unspecified
N64.4	Mastodynia
I20.9	Angina Pectoris, unspecified

**Table 8:** ICD-10-CM Billing Codes Used to Compile Cosmos Myalgia Pain Sub-Group

ICD-10-CM Code	Code Description
M79.1	Myalgia
M79.18	Myalgia, other site
M79.10	Myalgia, unspecified site

**Table 9:** ICD-10-CM Billing Codes Used to Compile Cosmos Headache Pain Sub-Group Headache includes 14 ICD-10-CM codes. Several representative codes are listed above.

ICD-10-CM Code	Code Description
R51	Headache
R51.9	Headache, unspecified
G44	Other headache syndromes
G44.8	Other specified headache syndromes
G44.59	Other complicated headache syndrome
G44.00	Cluster headache syndrome, unspecified
G43.C1	Periodic headache syndromes in child or adult, intractable
